# Acute ischemic stroke following Hump-nosed viper envenoming; first authenticated case

**DOI:** 10.1186/1477-9560-10-21

**Published:** 2012-09-20

**Authors:** Vijayabala jeevagan, Thashi Chang, Christeine Ariaranee Gnanathasan

**Affiliations:** 1 University medical unit, National hospital of Sri Lanka, Colombo, Sri Lanka; 2 Department of clinical medicine, Faculty of medicine, University of Colombo, Colombo, Sri Lanka

## Abstract

Hump-nosed pit viper (Genus Hypnale) is a medically important venomous snake in Sri Lanka and Southwestern India which causes significant morbidity and mortality. Envenoming of this snake results in hemostastic dysfunction, thrombotic microangiopathy, acute kidney injury and death. This case describes an authenticated first case of ischemic stroke in a 65 year old male following envenoming by H.hypnale in Sri Lanka.

## Introduction

**Figure 1:**
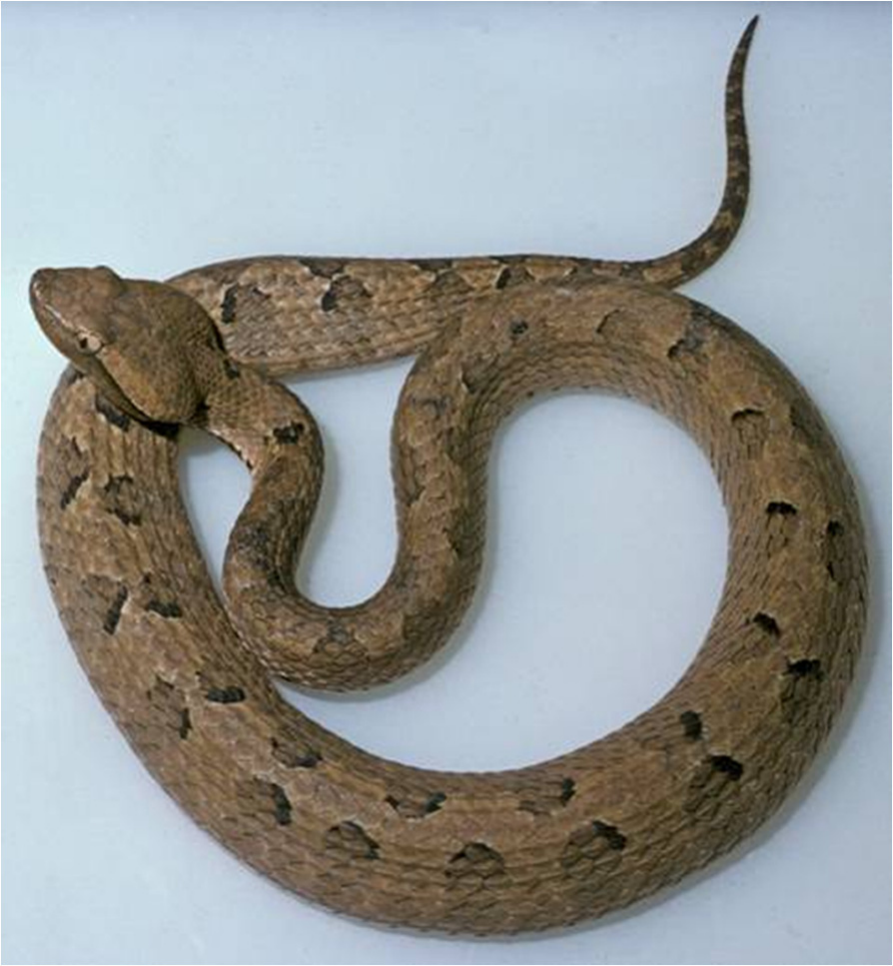
** Hump-nosed viper (*Hypnale hypnale*).**

Hump-nosed vipers (*Hypnale hypnale*) (Figure 1) are traditionally considered as moderately venomous snakes. It is the commonest cause of venomous snakebites in Sri Lanka accounting for 22 to 77% of all snakebites [1-3]. Varying degrees of local effects, hemostastic dysfunction, microangiopathic haemolysis, acute kidney injury and death have been reported following *H.hypnale* envenomation [1]. In the past decade several cases of ischaemic stroke following Russell viper and Saw scale viper envenoming have been reported [4,,^5^]. We report the first case of ischaemic stroke following *H.hypnale* envenoming.

## Case report

A 65-year-old farmer presented with pain and swelling of his foot following a hump-nosed viper bite. The culprit snake was killed and brought for identification. There was no evidence of systemic envenoming and the 20 minute-whole blood clotting time (20-WBCT) was normal. However, 24 hours later he developed sudden onset left-sided hemiparesis with a muscle power of 3/5, exaggerated tendon reflexes and an extensor plantar response on the left. This was followed by a declining urine output and the development of intractable hiccups. He has been on atorvastatin and aspirin for stable ischaemic heart disease. He did not have hypertension or diabetes mellitus. He has not smoked tobacco for 10 years.

**Figure 2:**
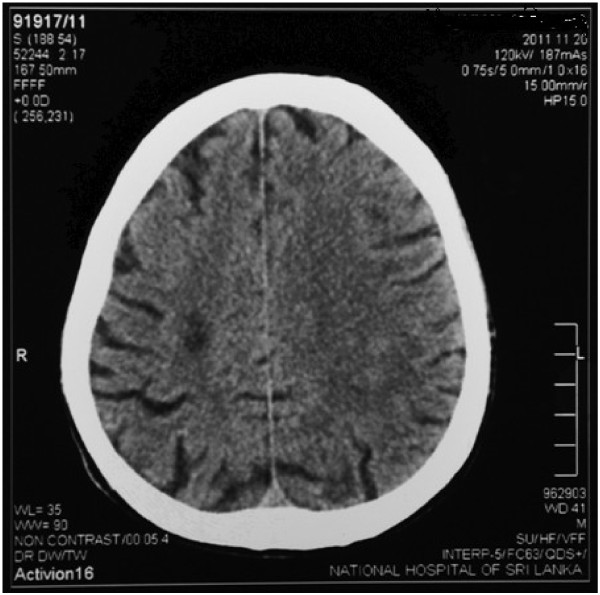
Computerized tomography picture showing a right sided deep parietal infarct.

A plain CT scan of the brain (Figure 2) showed an acute infarct in right deep parietal region. His hemoglobin was 13.2 g/dl, white cell count was 15.6 x 10^9^/l (neutrophils 80%) and the platelet count was 154 x 10^9^/l. The 20-WBCT, prothrombin time, thrombin time and partial thromboplastin time with kaolin tests were monitored periodically during hospital stay and all found to be normal. His D-dimer level was 750 ng/ml (110-250 ng/ml). His electrocardiogram (ECG), transthoracic echocardiogram, lipid profile, fasting plasma glucose, duplex scan of carotids and thrombophilic screening (including anticardiolipin antibody, lupus anticoagulation and genetic screening for inherited thrombophilias) were normal. However, his serum creatinine progressively increased to 348 μmol/l. His highest blood urea was 20.6 mmol/l(2.9-8.2 mmol/l). He had no features of dehydration and repeated fluid challenges failed to restore urine out put.

He was treated with supportive care. His urine out put gradually improved without renal replacement therapy and the serum creatinine returned to normal after 5 days. He was discharged home a week later with an out-patient plan for stroke rehabilitation.

## Discussion

Cerebral infarction following snake bite envenoming is uncommon. Arterial thrombotic complications including is chaemic strokes have been reported following Russel viper envenoming in Sri Lanka [6], but not following hump-nosed viper bites. Our patient developed cerebral infarction 48 hours following hump-nosed viper bite. When considering his age and history of ischemic heart disease it could be a coincidental event. However the presence of potent procoagulants in hump-nosed pit viper venom, made us to consider a possible etiological role of hump-nosed viper envenoming for the cerebral infarction. Furthermore the onset of acute ischemic stroke coincided with the onset of acute kidney injury, which indicated that active venom was present in substantial amounts to damage his kidneys. Elevated D-dimer in our patient is compatible with increased activation of the procoagulant and fibrinolytic mechanisms. In our patient pre existing atherosclerosis might act as a predisposing factor in the mechanism of H.hypnale venom induced thrombotic occlusion.

Clinical and preclinical studies have shown that *H.hypnale* venom possess strong procoagulant, haemorrhagic and necrotic activities through the presence of phospolipase A2, hyaluronidase, L-amino acid oxidase, thrombin-like enzymes, arginine esterase and various proteases [7-10]. Arginine esterase hydrolase, a thrombin-like enzyme activates clotting factors V and X of the intrinsic and common coagulation pathways leading to thrombin formation and intravascular deposition of fibrin causing vascular occlusion. Furthermore, direct action of the venom on vascular endothelial cells causes vasospasm that may play a role in arterial occlusion [11,,^12^]. Phospholipase A2 is a potent vasodilator that can cause acute hypotension and watershed brain infarctions, but this was not the case in our patient.

It is therefore plausible that the hump-nosed viper envenoming was the primary mechanism of vascular occlusion and stroke in our patient, but systematic studies are required to establish a cause-effect relationship. It is curious that although hump-nosed viper venom possesses the potential for vascular occlusion, there were no previous reports of stroke following envenoming. It is possible that stroke following hump-nosed viper bite is a result of a multifactorial process that depends on several factors such as the dose and composition of the venom and an underlying predisposition such as critical cerebral atherosclerosis, or that minor strokes in clinically silent areas of the brain were unrecognized.

To date, there is no effective antivenom for the treatment of hump-nosed viper envenoming. However, if strokes are increasingly recognized following hump-nosed viper bites, an antivenom will be required to prevent thrombotic complications as was shown with a substantial reduction of thrombotic complications in patients treated with antivenom following *Bothrops lanceolatus* envenoming [13]. This case report is intended to increase the vigilance for arterial thrombosis following hump-nosed viper envenomation.

## Consent

Written informed consent was obtained from the patient for publication of this Case report and accompanying images. A copy of the written consent is available for review by the Editor-in-Chief of this journal.

## Competing interests

The authors declare that they have no competing interests.

## Authors’ contributions

All authors were involved in the management of the patient. VJ prepared the initial manuscript. AG and TC were involved in clinical decision making and they reviewed the manuscript with regard to scientific data. All authors read and approved the final manuscript.

## References

[B1] Ariaratnam CA Thuraisingam V Kularatne SA Sheriff MH Theakston RD de Silva A Warrell DA Frequent and potentially fatal envenoming by hump-nosed pit vipers (Hypnale hypnale and H. nepa) in Sri Lanka: lack of effective antivenom Trans R Soc Trop Med Hyg 2008 102 11 1120 1126 10.1016/j.trstmh.2008.03.023 18455743

[B2] De Silva A Snake bite in Anuradhapura District The Snake 1981 13 117 130

[B3] Seneviratne SL Opanayaka CJ Ratnayake NS Kumara KE Sugathadasa AM Weerasuriya N Wickrama WA Gunatilake SB de Silva HJ Use of antivenom serum in snake bite: a prospective study of hospital practice in the Gampaha district Ceylon Med J 2000 45 2 65 68 11051703 10.4038/cmj.v45i2.8003

[B4] Gawarammana I Mendis S Jeganathan K Acute ischemic strokes due to bites by Daboia russelii in Sri Lanka - first authenticated case series Toxicon 2009 54 4 421 428 Epub 2009 May 20 10.1016/j.toxicon.2009.05.006 19463846

[B5] Bashir R Jinkins J Cerebral infarction in a young female following snake bite Stroke 1985 16 328 330 10.1161/01.STR.16.2.328 3975973

[B6] Ameratunga B Middle cerebral occlusion following Russel’s viper bite J Trop Med Hyg 1972 75 5 95 97 5032231

[B7] Kalana M Hodgson WC Nicki K O'Leary MA Indika Gawarammana, and Geoffrey K Isbister. The in vitro toxicity of venoms from South Asian Hump-nosed pit vipers (Viperidae: Hypnale) J Venom Res 2011 2 E17 PMC3114463 21677795

[B8] de Silva A Wijekoon ASB Jayasena L Abeysekera CK Cheng-Xin B Button RA Warrell DA Haemostatic dysfunction and acute renal failure following envenoming by Merrem's hump-nosed viper (Hypnale hypnale) in Sri Lanka: first authenticated case Trans R Soc Trop Med Hyg 1994 88 209 212 10.1016/0035-9203(94)90301-8 8036678

[B9] Premawardena AP Seneviratne SL Gunatilake SB de Silva HJ Excessive fibrinolysis: the coagulopathy following Merrem's hump-nosed viper (Hypnale hypnale) bites Am J Trop Med Hyg 1998 58 6 821 823 9660472 10.4269/ajtmh.1998.58.821

[B10] Tan CH Sim SM Gnanathasan CAI Fung SY Ponnudurai G Pailoor JV Tan NH Enzymatic and toxinological activities of Hypnale hypnale (hump-nosed pit viper) venom and its fractionation by ion exchange high performance liquid chromatography J. Venom. Anim. Toxins incl. Trop. Dis 2011 17 4 Botucatu 10.1590/S1678-91992011000100002

[B11] Murthy JM Kishore LT Naidu KS Cerebral infarction after envenomation by viper J Comput Assist Tomogr 1997 21 1) 35 37 9022766 10.1097/00004728-199701000-00007

[B12] Bashir R Jinkins J Cerebral infarction in a young female following snake bite Stroke 1985 16 2 328 330 10.1161/01.STR.16.2.328 3975973

[B13] Thomas L Tyburn B Ketterlé J Biao T Mehdaoui H Moravie V Rouvel C Plumelle Y Bucher B Canonge D Marie-Nelly CA Lang J Prognostic significance of clinical grading of patients envenomed by Bothrops lanceolatus in Martinique. Members of the Research Group on Snake Bite in Martinique Trans R Soc Trop Med Hyg 1998 92 5 542 545 10.1016/S0035-9203(98)90907-5 9861375

